# Effects of Low-Calorie Nutrition Claim on Consumption of Packaged Food in China: An Application of the Model of Consumer Behavior

**DOI:** 10.3389/fpsyg.2021.799802

**Published:** 2022-01-28

**Authors:** Zeying Huang, Haijun Li, Pei Wang, Jiazhang Huang

**Affiliations:** ^1^Institute of Food and Nutrition Development, Ministry of Agriculture and Rural Affairs, Beijing, China; ^2^School of Information & Intelligence Engineering, University of Sanya, Sanya, China; ^3^School of Public Health, Southeast University, Nanjing, China

**Keywords:** nutrition claim, low-calorie products, nutrition labeling, packaged food, model of consumer behavior

## Abstract

More and more packaged products in China have been labeled as low-calorie products since the official implementation of nutrition claims in 2007. But little was known about the impact of such claims on the Chinese consumption of low-calorie food on the background of increasing rates of obesity among the Chinese population. This study sought to fill the gap by applying a consumer behavior model to a nationally representative online survey by means of structural equation modeling. The findings revealed that nutrition claims significantly affect the consumption of low-calorie products. Specifically, marketing stimulus on low-calorie products first affected consumer psychology, then consumer decision-making, and finally consumer responses. Despite the significant role of consumer psychology and decision-making in consumption, consumers were susceptible to the influence of targeted marketing strategies for foods with a low-calorie claim. It is recommended that appropriate use of low-calorie nutrition claims by manufacturers and choices of low-calorie food by consumers according to their own needs should be encouraged.

## Introduction

Overweight and obesity are growing problems in China and have been linked to a variety of adverse health outcomes (e.g., diabetes, heart disease, and certain cancers). Report on Nutrition and Chronic Diseases in China (2020) illustrated that over 50% of adults were overweight or obese in 2019, an increase of at least 8% from 2012 (Central People’s Government of the People’s Republic of China, 2020). Given that the calorie intake exceeding the calorie expenditure is a strong contributor to overweight or obesity (Blundell, 1975), consumers are increasingly concerned with the amount of calories from food (Zhang et al., 2008).

Nutrition claim, a nutrition labeling first advocated by the Codex Alimentarius Commission, refers to any representation which states, suggests, or implies that a food has particular nutritional properties (e.g., fat free, low in sugars, high protein, and source of calcium) (Codex Alimentarius Commission, 1985). Many countries have adopted Codex recommendations and regulated the use of nutrition claims (Franco-Arellano et al., 2018). For improvement in diet quality, China implemented *Food Nutrition Labeling Management Standards* in 2007, introducing voluntary nutrition claims to the country for the first time. The low-calorie claim, a form of nutrient content claim, can be used on prepackaged food of China when calorie is less than 170 kJ/100 g for solids or 80 kJ/100 ml for liquids, according to *General Rules of National Prepackaged Food Nutrition Labels* (GB 28050-2011) (Ministry of Health of the PRC, 2011), thus some food or drink products (e.g., low-calorie bread, low-calorie soda water) have been labeled as “low in calorie” by manufacturers. Therefore, identifying the impact of such a claim on the consumption of low-calorie packaged products is of importance to understand whether this claim is helpful to reduce the calorie intake of consumers.

Previous studies on the nutrition claim mainly focused on the influence of regulation concerning nutrition claims on food choice of consumers (Mathios, 1998; Stranieri et al., 2010) and preference of consumers for food labeled with nutrition claims (Wezemael et al., 2014; Klopčič et al., 2019). It remained unknown whether and how nutrition claims and other key factors impact low-calorie food consumption of consumers, although a few studies assessed the impact of nutrition claims on perceptions (Benson et al., 2019), attitudes (Garretson and Burton, 2000), and food choices of consumers (Oostenbach et al., 2019). In China, some studies investigated the objective understanding of Chinese residents toward nutrition claims (Song et al., 2015) and nutrients displayed on nutrition claims in China (Wang et al., 2011), but few studies focused on the impact of nutrition claims.

This study aims to achieve a better understanding of how a low-calorie-related claim influences consumption of prepackaged food by Chinese residents. Structural equation modeling (SEM) was utilized to establish possible linkages by employing the model of consumer behavior as an analytical framework. To the best of our knowledge, it is the first study in China to empirically investigate the consumption of foods with a low-calorie claim from the perspective of consumers. The findings will assist policymakers in achieving the public health goal that increases healthier food consumption.

## Hypotheses

The model of consumer behavior used in this work is proposed by Professor Philip Kotler (Kotler and Armstrong, 2011), stems from the stimulus-response (S-R) model, and is a commonly used theoretical model describing the real and reasonable consumption process.

In contrast to other theories, the model of consumer behavior takes marketing stimulus as the only external element and uses consumer psychology, consumer decision-making, and consumer responses as internal elements. In other words, the model emphasizes consumption behavior in the process of consumer psychology, consumer decision-making, and consumer responses under marketing stimuli. Generally, foods with specific nutrition claims provide opportunities for product differentiation based on a health-related positioning, as compared with those without such claims (Verbeke et al., 2009). Nutrition claim is known as a marketing tool to make the products appear healthier resulting from the halo effect (Story and French, 2004; Barreiro-Hurlé et al., 2009), so manufacturers attempt to increase the exposure of consumers to nutrition claims through marketing and advertising (Andrews et al., 2000). Thus, the model of consumer behavior can be applied to identify the relationships and pathways between nutrition claims and consumers’ behavior.

**FIGURE 1:**
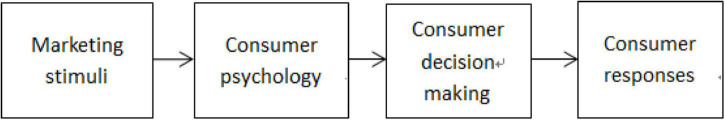
Model of consumer behavior. Source: Kotler and Armstrong (2011).

The proposed conceptual model in Figure 1 suggests that marketing stimuli first influence consumer psychology, then consumer decision-making, and finally consumer responses. Marketing stimuli consists of four P’s, namely, product, price, place, and promotion, which, in this study, respectively, refer to the food labeled with a low-calorie claim, the amount of money customers must pay for such food, the channels through which consumers have access to such food, and the activities that advertise the merits of the claim to encourage consumer purchase.

Consumer psychology refers to the psychological activities of consumers when they seek, choose, purchase, use, evaluate, and dispose of products. The psychosocial-anthropological approach indicates that there are four types of physiological effects among consumers, namely, herd mentality, the mind of difference, the mind of rivalry, and the practical mind (Katona, 1967). Specifically, herd mentality refers to the phenomenon that an individual’s ideas and behaviors involuntarily or unconsciously are consistent with those of the majority due to group guidance or pressure; the mind of difference means that individuals pursue originality and distinctness in consumption processes; the mind of rivalry is an individual’s consumption desire beyond one’s actual income level; the practical mind is primarily characterized by attention to the actual use value of goods. Most previous research on the effect of marketing- or advertising-driven strategies of manufacturers on consumer psychology concludes that marketing produces a positive influence (Foxall, 1986; Aaker et al., 2000). Hence, hypothesis 1 is proposed:

H1. Marketing of foods with a low-calorie nutrition claim has a positive effect on consumer psychology.

Consumer decision-making consists of cognition, experience, evaluation, and purchase (Kotler and Armstrong, 2011). Several empirical studies have found that consumer psychology could pose an influence on consumer decision-making (Foxall, 2016; Ebert, 2017). Hence, hypothesis 2 is proposed:

H2. Consumer psychology influences the decision-making process regarding foods with a low-calorie nutrition claim.

Consumer responses, generated after the process of decision-making, are composed of product selection, brand selection, purchase timing, and purchase quantity (Kotler and Armstrong, 2011). Specifically, product selection refers to the behavior of consumers in choosing a product; brand selection refers to the choice of consumers for the product brand; purchase timing refers to the time when consumers buy the product; and purchase quantity refers to how many/much products consumers buy. Prior studies have identified the links between consumer decision-making and consumer responses (Fan and Xiao, 1998; Loureiro and Mccluskey, 2000). Hence, hypothesis 3 is proposed:

H3. Consumer decision-making impacts consumer responses to foods with a low-calorie nutrition claim.

## Materials and Methods

### Participants and Procedure

**FIGURE 2:**
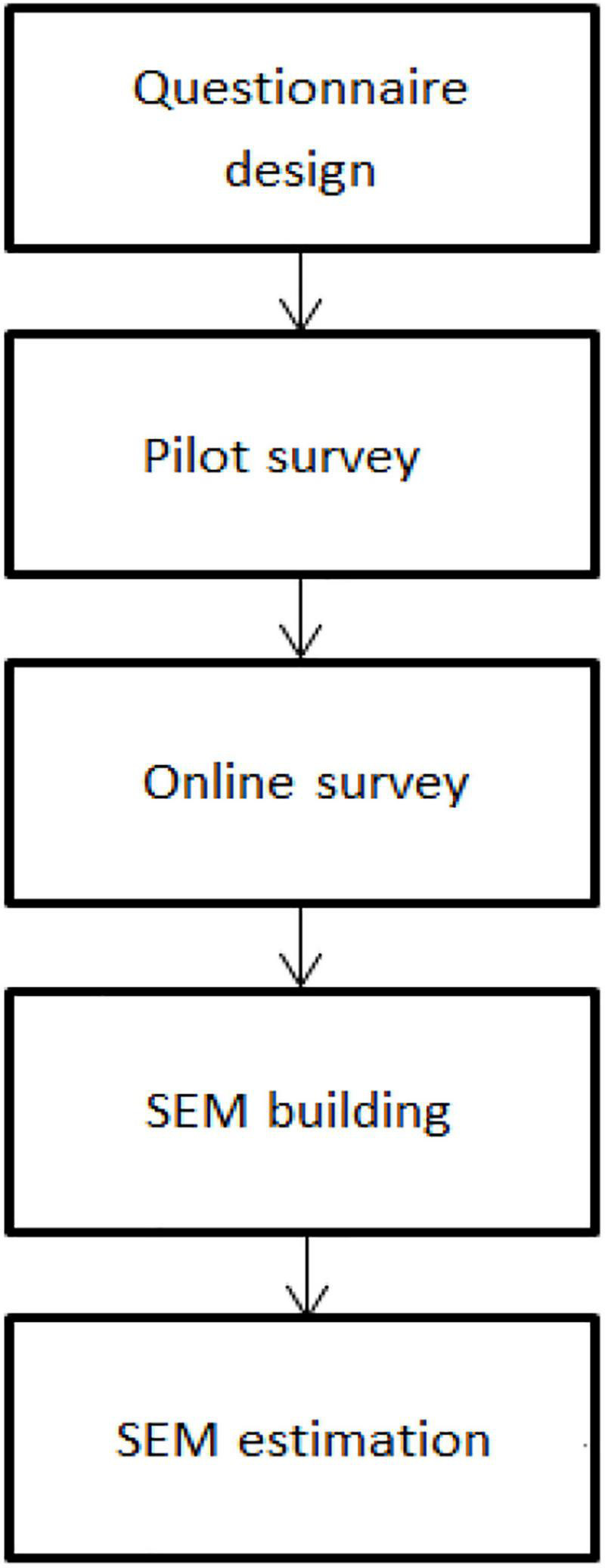
The experimental design.

With respect to the model of consumer behavior, the four latent variables, namely, marketing stimuli, consumer psychology, consumer decision-making, and consumer responses could be measured by scale items designed based on a review of previous literature (Priester, 2010; Devika et al., 2020). Figure 2 illustrated the general experimental design process. The data were collected using a self-administered questionnaire consisting of socio-demographic information and 20 scale items (see “Supplementary Material’’). Each item was answered on a 5-point Likert scale (1 = strongly disagree to 5 = strongly agree), and 80 residents were randomly selected in Beijing by a pretest on October 21, 2020. As a company specializing in data collection *via* online surveys, Wenjuanxing platform^1^ has a sample pooling of 2.6 million potential respondents uniformly distributed by gender, age groups, and regions. Paid data collection service is provided by the Wenjuanxing platform for sending questionnaires to target samples and ensuring the validity of questionnaire information. From November 10, 2020, to December 28, 2020, the Wenjuanxing platform was commissioned to obtain 930 valid survey samples from the sample pooling. First of all, a stratified sampling approach was used to randomly select 45 individuals from each province (i.e., autonomous region, municipality) of China to fill out the online questionnaire, and then cross-checking was conducted to eliminate invalid questionnaires due to lack of information and implausible answers. Finally, a total of 930 valid samples (i.e., 30 samples × 31 provinces) were generated for analysis.

### Measures

Propositions that connect endogenous variables with exogenous variables were analyzed using SEM, which provides a dependable framework for testing differences among groups on latent variables (i.e., constructs, factors) (Graham and Roberto, 2016). SEM allows the creation of observable variables per construct, which does not require the split analysis and yields valid and clear inferences (Lei and Wu, 2007), thus results of the relationships among variables were reliable and neutral (Neale et al., 2016). In addition, SEM is capable of scrutinizing complex correlations and a range of hypotheses by immediately incorporating mean structures and group estimation (Al-Gahtani, 2016). Therefore, the hypotheses proposed above were made out by the SEM. Specifically speaking, all data analyses were conducted in two steps. First, the reliability analysis was performed using the SPSS 25.0 software to evaluate the stability and consistency of measured items. Second, the evaluation of goodness-of-fit indices for the proposed SEM and tests of hypotheses was conducted by means of moment structure analysis using the AMOS 21.0 software.

## Results

The final sample is nationally representative of the Chinese population in terms of socio-demographic characteristics. Table 1 below outlines the demographic features of 930 valid samples. More males were surveyed (56.77%), and respondents were mostly young adults, with those aged between 18 and 44 years comprising about 31.61% of the sample. The majority of respondents had high school education (31.29%) followed by junior high school degrees (30.43%). Among the respondents, most (27.31%) had an annual household income between ¥10,000 and 50,000 after tax, but the percentage was slightly higher than those with an income from ¥50,001 to 100,000.

**TABLE 1:** Socio-demographic characteristics of the sample (*n* = 930).

Sample characteristics	Option	Sample size	Percentage (%)
Gender	Male	528	56.77
	Female	402	43.23
Age	<18	168	18.06
	From 18 to 44	294	31.61
	From 45 to 59	275	29.57
	≥9.5	193	20.76
Education level	Primary school and below	127	13.66
	Junior high school	283	30.43
	High school	291	31.29
	College/Bachelor	198	21.29
	Postgraduate or above	31	3.33
Annual household income (after tax)	<10,000 Yuan	117	12.58
	From 10,000 Yuan to 50,000 Yuan	254	27.31
	From 50,001 Yuan to 100,000 Yuan	246	26.45
	From 100,001 Yuan to 150,000 Yuan	180	19.35
	From 150,001 Yuan to 200,000 Yuan	83	8.92
	>200,000 Yuan	50	5.38

*One US dollar is equal to 6.524 Chinese Yuan and One Euro is equal to 7.960 Chinese Yuan from November 10 to December 28, 2020.*

Table 2 presents the reactions of respondents toward the marketing of products by manufacturers with a low-calorie nutrition claim, together with consumer psychology, decision-making, and responses. The marketing strategy of products with a low-calorie claim mainly played a role with respect to product and place. More than half of the respondents (55.81%) had seen products with a low-calorie nutrition claim on sales, and nearly 50% had seen these products in multiple places. The respondents paid attention to such a claim with the herd mentality and practical mind rather than the mind of difference and rivalry. Up to 60.53% of the respondents indicated that they would follow their friends and relatives who read nutrition claims related to low calories. Also, as many as 64% would pay attention to the practical benefits of food with such claims. Few respondents had experience in reading nutrition claims or purchasing food with nutrition claims, whereas approximately 66 and 65% of them believed that the low-calorie nutrition claim could help make a healthy food choice and understand nutritional properties, respectively. Regarding consumer responses, the low-calorie claim shaped the choices of respondents among different kinds of foods, among different brands of similar foods, among different food package sizes, and prompted impulse purchase of low-calorie foods.

**TABLE 2:** Description of latent variables and summary statistics.

Latent variables	Scale items	Strongly disagree		Disagree		Neither agree nor disagree		Agree		Strongly agree	
						
		N	%	N	%	N	%	N	%	N	%
Marketing stimuli	I have seen food with low-calorie nutrition claim on sales.	21	2.26	110	11.83	280	30.11	374	40.22	145	15.59
	I know that the price of food with low-calorie nutrition claim is affordable.	108	11.61	288	30.97	376	40.43	138	14.84	20	2.15
	I have seen food with low-calorie nutrition claim sold in many places.	31	3.33	153	16.45	300	32.26	336	36.13	110	11.83
	I have seen the food with low-calorie nutrition claim on promotion.	64	6.88	301	32.37	338	36.34	178	19.14	49	5.27
Consumer psychology	I would follow my friends and relatives’ example if they all read low-calorie nutrition claim when shopping.	24	2.58	85	9.14	258	27.74	455	48.92	108	11.61
	I would read low-calorie nutrition claim even if none of my friends and relatives did it when shopping.	36	3.87	223	23.98	380	40.86	226	24.30	65	6.99
	I would buy the food with low-calorie nutrition claim which is beyond my factual income.	83	8.92	291	31.29	352	37.85	176	18.92	28	3.01
	I would pay attention to the actual benefits of food with low-calorie nutrition claim.	15	1.61	49	5.27	271	29.14	446	47.96	149	16.02
Consumer decision making	I believe low calorie nutrition claim helps make healthy food choice.	14	1.51	70	7.53	231	24.84	453	48.71	162	17.42
	I believe low calorie nutrition claim helps understand nutritional properties of food.	17	1.83	64	6.88	247	26.56	428	46.02	174	18.71
	I have read low calorie nutrition claim when shopping.	29	3.12	222	23.87	340	36.56	263	28.28	76	8.17
	I have bought foods with low calorie nutrition claim when shopping	14	1.51	135	14.52	397	42.69	289	31.08	95	10.22
Consumer responses	I have made choices among different kinds of foods through low-calorie nutrition claim.	31	3.33	210	22.58	334	35.91	283	30.43	72	7.74
	I have made choices among different brands of similar foods through low-calorie nutrition claim.	41	4.41	190	20.43	353	37.96	276	29.68	70	7.53
	I have seized the moment to buy foods with low-calorie nutrition claim.	42	4.52	232	24.95	376	40.43	228	24.52	52	5.59
	I have made choices among different amounts of food through low-calorie nutrition claim.	73	7.85	221	23.76	369	39.68	194	20.86	73	7.85

### Discriminant Validity Analysis

The interrelationships among constructs affecting the behavior of consumers were assessed using Pearson’s correlation test, and statistically significant (*p* < 0.05) positive correlations were reported (as described in Table 3). Discriminant validity was also assessed by examining the average variance extracted estimates (AVE). The discriminant validity analysis evaluates the extent to which the items are not theoretically correlated. The data did not have any problems of discriminant validity because the value of the square root of AVE was greater than its correlation with other constructs (Kline, 2005).

**TABLE 3:** Factor correlations and discriminant validity.

Factors	Marketing stimuli	Consumer psychology	Consumer decision making	Consumer responses
Marketing stimuli	[0.792]			
Consumer psychology	0.494**	[0.787]		
Consumer decision making	0.451*	0.913***	[0.705]	
Consumer responses	0.333*	0.674*	0.739**	[0.733]

Values in brackets [] indicate the square root of AVEs. A significance level is shown at ***p < 0.001, **p < 0.01, and *p < 0.05. Diagonals represent the square root of the average variance extracted, while the other entries represent the squared correlations.

### Testing the Fit of the Model

An exploratory factor analysis (EFA) was employed to examine the reliability and validity of the measurement model. The suitability of data was examined by using the Kaiser-Meyer-Olkin (KMO) sampling adequacy test and Bartlett’s Test of Sphericity (BTS). As seen in Table 4, the sample is suitable to conduct EFA, owing to the statistically significant BTS value and KMO value (Kaiser, 1974). Also, the consistency of items of all constructs was examined by the composite reliability (CR) test. To evaluate the level to which the items were theoretically associated with each other, the convergent validity test was implemented by using AVE and item loadings (Anderson and Gerbing, 1988). Further empirical results revealed that all AVE values surpassed 0.50 for each construct, and this indicated that the latent constructs retained a minimum of 50% of the variance. The values of CR and Cronbach’s α exceeded 0.70 in all four constructs through a reliability analysis test (Nunnally, 1978), indicating that the sample was valid and reliable.

**TABLE 4:** Factor loadings and convergent validity results.

Variables	Scale items Code	Scale items	Standard Loadings	AVE	Composite reliability	Cronbach’ s α
Marketing stimuli	X1	I have seen food with low-calorie nutrition claim on sales.	0.506	0.627	0.703	0.816
	X2	I know that the price of food with low-calorie nutrition claim is affordable.	0.641			
	X3	I have seen food with low-calorie nutrition claim sold in many places.	0.562			
	X4	I have seen the food with low-calorie nutrition claim on promotion.	0.554			
Consumer psychology	X5	I would follow my friends and relatives’ example if they all read low-calorie nutrition claim when shopping.	0.588	0.619	0.734	0.853
	X6	I would read low-calorie nutrition claim even if none of my friends and relatives did it when shopping.	0.591			
	X7	I would buy the food with low-calorie nutrition claim which is beyond my factual income.	0.501			
	X8	I would pay attention to the actual benefits of food with low-calorie nutrition claim.	0.516			
Consumer decision making	X9	I believe low-calorie nutrition claim helps make healthy food choice.	0.661	0.597	0.769	0.826
	X10	I believe low-calorie nutrition claim helps understand nutritional properties of food.	0.635			
	X11	I have read low-calorie nutrition claim when shopping.	0.718			
	X12	I have bought foods with low-calorie nutrition claim when shopping.	0.643			
Consumer responses	X13	I have made choices among different kinds of foods through low-calorie nutrition claim.	0.729	0.537	0.792	0.899
	X14	I have made choices among different brands of similar foods through low-calorie nutrition claim.	0.755			
	X15	I have seized the moment to buy foods with low-calorie nutrition claim.	0.760			
	X16	I have made choices among different amounts of food through low-calorie nutrition claim.	0.752			

*Rotation technique: Promax; extraction technique: maximum likelihood; total variance elucidated: 59.05%; Bartlett’s test of sphericity: χ2 = 5,901.666, p < 0.001; Kaiser-Meyer-Olkin measure of sampling adequacy: 0.884 (p < 0.001).*

### Structural Equation Modeling Estimation and Hypothesis Testing

**FIGURE 3:**
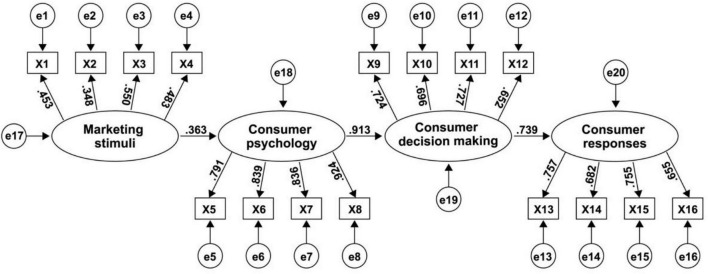
Structural equation modeling results. Comparative fit index = 0.850; goodness-of-fit index = 0.887; root mean square error of approximation = 0.075; degrees of freedom = 136; chi-square = 1,029.143; X1–X16 is the scale items code and e1–e20 is statistical error of four latent variables and 16 scale items.

All hypotheses proposed were examined after the validity and reliability of the measures were attained. Figure 3 shows the estimation result, and Table 5 displays the goodness-of-fit indices for the model. Each fitting index value [standard chi-square (SCS) = 2.199, comparative fit index (CFI) = 0.950, incremental fit index (IFI) = 0.951, goodness-of-fit index (GFI) = 0.987, AGFI = 0.957, root mean square error of approximation (RMSEA) = 0.075, non-normalizing fitting index (NNFI) = 0.928, norm fitting index (NFI) = 0.927] outperformed the respective threshold value, signifying that the model was able to fit all data satisfactorily (Byrne, 1994; Thompson, 2004; Kline, 2005).

**TABLE 5:** Structural equation modeling fitting.

Goodness-of-fit indices	Fitting index values	Fitting
Standard chi – square (SCS)	2.199	<3, good
Comparative fit index (CFI)	0.950	>0.9, good
Incremental fit index (IFI)	0.951	>0.9, good
Goodness-of-fit Index (GFI)	0.987	>0.9, good
Adjusted goodness-of-fit index (AGFI)	0.957	>0.9, good
Root mean square error of approximation (RMSEA)	0.075	<0.08, good
Non-normalizing fitting index (NNFI)	0.928	>0.9, good
Norm fitting index (NFI)	0.927	>0.9, good

As expected, all null hypotheses were supported at the statistical significance level of 0.01 (Table 6). Marketing stimuli had significant effects on consumer psychology, and the path coefficient was 0.363. Likewise, consumer psychology posed a significant impact on consumer decision-making, with the largest coefficient (0.913) out of the three relationships, which was followed by the path coefficient of consumer decision-making on consumer responses.

**TABLE 6:** Test results of the hypothesis.

Hypothesized paths	Normalized path coefficient	*T*-value	*P*-value	Accepted
H1: Marketing stimuli→Consumer psychology	0.363	1.382	0.007	Yes
H2: Consumer psychology→Consumer decision making	0.913	3.411	0.005	Yes
H3: Consumer decision making→Consumer responses	0.739	3.835	0.008	Yes

## Discussion

The present study has demonstrated two key strengths. On one hand, the model of consumer behavior offered a conceptual framework for exploring how the marketing strategy of food by manufacturers with a low-calorie nutrition claim influenced consumption behavior. On the other hand, this study considered consumer behavior as a process of transformation from individual psychology to behavior, which could identify whether the behaviors of Chinese consumers were rational or not in the context of marketing. As for methodological strength, the application of SEM was helpful to reveal interrelationships among constructs affecting consumer behavior. However, several limitations need to be acknowledged and addressed in future studies. First, the intrinsic factors in the model of consumer behavior did not take into account the socio-demographic characteristics of consumers, such as gender, age, occupation, and education level. Second, the model did not regard consumer cognition (Drugǎu-Constantin, 2019; Pocol et al., 2021), attitude (Graessley et al., 2019), and loyalty (Hollowell et al., 2019) as factors, despite the proven role of these factors in shaping the consumption behavior of individuals. Third, this study lacked an examination of satisfaction of consumers with consumption experience, which was yet proved an integral part of consumer behavior (Reibstein et al., 1975; Gounaris et al., 2007). Fourth, respondents may make wrong answers in the self-filled questionnaire concerning consumer psychology, and neuroscience techniques such as brain waves and eye movement tracking technologies, which have been thought to overcome such problems (Drugǎu-Constantin, 2019), should be considered to use in the future study. The last possible limitation is related to the small sample size analyzed in comparison with the Chinese large population, which hardly guaranteed that our findings above could be replicated within relevant studies, thus we call for a larger sample included.

### Association Between Marketing Stimuli and Consumer Psychology

The research result coincided with the marketing theory of 4P’s and findings of existing studies (Foxall, 1986; Aaker et al., 2000), which suggested that current marketing strategies of food with low-calorie nutrition claims on the Chinese market could somewhat exert influence on consumer psychology. To be specific, manufacturers focused on the development of food nutrition function, product pricing, sales channel establishment, and product publicity, all of which were able to cause the psychological reaction of consumers and to increase their desire for low-calorie food (Wing et al., 1991; O’Neil and Jarrell, 1992). However, the marketing of food products with low-calorie claims played a minor role, which may be due to the recent scandals of “low calorie” misrepresentation on food packages in China and the concern of consumers about being misled. Meanwhile, it reflected that consumers may know little about the principles of nutrition claim usage.

### Association Between Consumer Psychology and Consumer Decision-Making

This study found convincing evidence of a statistically significant link between consumer psychology and consumer decision-making, consistent with the results of existing studies (Foxall, 2016; Ebert, 2017).

In detail, consumer psychology toward the food with low-calorie nutrition claims, such as herd mentality, the mind of difference, the mind of rivalry, and practical mind, was found to likely affect their trust and use of low-calorie nutrition claim. It implied that marketing stimuli of manufacturers were likely to exert an obvious effect on consumer decision-making through shaping consumer psychology. That is, the decision of consumers to purchase and consume low-calorie products with help of nutrition claims was significantly influenced by marketing stimuli and is also mediated by consumer psychology.

### Association Between Consumer Decision-Making and Consumer Responses

The study confirmed previous findings that consumer responses could be influenced by how a consumer reaches a decision (Kim and John, 2008; Aggarwal and Agarwal, 2015). Although the influence was not the strongest, consumers’ decision-making involving individuals’ trust (Dabija et al., 2018; Meilhan, 2019; Popescu and Ciurlǎu, 2019) on low-calorie nutrition claims, reading the claim, and purchasing related products seemed to have a positive impact on consumers’ product selection (Miricǎ, 2019), brand selection, purchase timing, and purchase quantity for products with low-calorie claims. This link was expected to increase the purchase and intake of healthy foods by consumers and eventually contribute to improved health status.

## Conclusion

The study highlights the importance of understanding the effects of low-calorie nutrition claims on the consumption of packaged food in China. The model of consumer behavior, as a theoretical and empirical framework, was proved to be possibly suitable for explaining the use of nutrition claims by Chinese residents. The findings suggested that marketing of products by manufacturers with low-calorie claims first affects consumer psychology and then, exerts a positive impact upon consumer decision-making, which ultimately determines consumer responses. To sum up, the application of low-calorie nutrition claims by manufacturers is positively associated with the increasing consumption rate of low-calorie food among the residents, which provides a theoretical basis for adult obesity intervention by using low-energy nutrition claim in China.

To increase the access to and the use of low-calorie nutrition claims, the following policy recommendations are offered: (1) manufacturers should be guided to adopt appropriate and reasonable marketing techniques to promote low-calorie foods; (2) public propaganda should be widely carried out to educate consumers to choose low-calorie food according to their physical conditions and occupational needs; and (3) the regulation and supervision of nutrition claim use by manufactures should be strengthened through authoritative nutritional guidelines and standards, resulting in social trust on nutrition claims.

## Data Availability Statement

The original contributions presented in the study are included in the article/Supplementary Material, further inquiries can be directed to the corresponding author.

## Ethics Statement

Ethical review and approval was not required for the study on human participants in accordance with the local legislation and institutional requirements. Written informed consent was obtained from all participants.

## Author Contributions

ZH contributed to conceptualization, original draft preparation, and methodology. HL conducted statistical analysis. PW performed data cleaning. JH carried out review and editing. All authors contributed to the article and approved the submitted version.

## Conflict of Interest

The authors declare that the research was conducted in the absence of any commercial or financial relationships that could be construed as a potential conflict of interest.

## Publisher’s Note

All claims expressed in this article are solely those of the authors and do not necessarily represent those of their affiliated organizations, or those of the publisher, the editors and the reviewers. Any product that may be evaluated in this article, or claim that may be made by its manufacturer, is not guaranteed or endorsed by the publisher.

## Footnotes

1
https://www.wjx.cn

## References

[B1] Aaker J. L. Brumbaugh A. M. Grier S. A. (2000). Nontarget markets and viewer distinctiveness: the impact of target marketing on advertising. *J. Consum. Psychol.* 9 127–140. 10.1207/S15327663JCP0903_1

[B2] Aggarwal P. Agarwal M. (2015). Linear versus step-function decision making: the moderating role of relationship norms on consumer responses to brand transgressions. *Rev. Mark. Res.* 12 207–232. 10.1108/S1548-643520150000012008

[B3] Al-Gahtani S. S. (2016). Empirical investigation of e-learning acceptance and assimilation: a structural equation model. *Appl. Comput. Inf.* 12 27–50. 10.1016/j.aci.2014.09.001

[B4] Anderson J. C. Gerbing D. W. (1988). Structural equation modeling in practice: a review and recommended two-step approach. *Psychol. Bull.* 103 411–423. 10.1037/0033-2909.103.3.411

[B5] Andrews J. C. Burton S. Netemeyer R. G. (2000). Are some comparative nutrition claims misleading? The role of nutrition knowledge, ad claim type and disclosure conditions. *J. Advert.* 29 29–42. 10.1080/00913367.2000.10673615

[B6] Barreiro-Hurlé J. Gracia A. de-Magistris T. (2009). Market implications of new regulations: impact of health and nutrition information on consumer choice. *Span. J. Agric. Res.* 7 257–268. 10.5424/sjar/2009072-417

[B7] Benson T. Lavelle F. Mccloat A. Mooney E. Bucher T. Egan B. (2019). Are the claims to blame? A qualitative study to understand the effects of nutrition and health claims on perceptions and consumption of food. *Nutrients* 11:2058. 10.3390/nu11092058 31480787 PMC6769963

[B8] Blundell J. E. (1975). Anorexic drugs, food intake and the study of obesity. *Int. J. Food Sci. Nutr.* 29 5–18. 10.3109/09637487509143887

[B9] Byrne B. M. (1994). *Structural Equation Modeling with EQS and EQS/Windows: Basic Concepts, Applications, and Programming.* Militas, CA: Sage.

[B10] Central People’s Government of the People’s Republic of China (2020). *Report on Nutrition and Chronic Diseases in China.* Available online at: http://www.gov.cn/xinwen/2020-12/24/content_5572983.htm (accessed October 1, 2021)

[B11] Codex Alimentarius Commission (1985). *Codex Guidelines on Nutrition Labeling*. CACGL 2-1985. Rome: Codex Alimentarius Commission.

[B12] Dabija D.-C. Bejan B. M. Grant D. (2018). The impact of consumer green behaviour on green loyalty among retail formats: a romanian case study. *Morav. Geogr. Rep.* 26 173–185. 10.2478/mgr-2018-0014

[B13] Devika R. Harikrishna M. Anjaneyulu M. V. L. R. (2020). Influence of psychological factors in mode choice decision making: a structural equation modeling approach. *Transp. Res. Proc.* 48 2821–2830. 10.1016/j.trpro.2020.08.236

[B14] Drugǎu-Constantin A. (2019). Is consumer cognition reducible to neurophysiological functioning? *Econ. Manag. Financ. Mark.* 14 9–15. 10.22381/EMFM14120191

[B15] Ebert S. (2017). Consumer psychology insights and their use for operational book marketing. *Exp. J. Bus. Manag*. 5 91–97.

[B16] Fan J. X. Xiao J. J. (1998). Consumer decision-making styles of young-adult Chinese. *J. Consum. Aff*. 32 275–294. 10.1111/j.1745-6606.1998.tb00410.x

[B17] Foxall G. R. (1986). Theoretical progress in consumer psychology: the contribution of a behavioural analysis of choice. *J. Econ. Psychol.* 7 393–414. 10.1016/0167-4870(86)90030-9

[B18] Foxall R. G. (2016). *Consumer Choice as Decision: Micro-Cognitive Psychology.* London: Palgrave Macmillan.

[B19] Franco-Arellano B. Labonté M. Bernstein J. T. L’Abbé M. R. (2018). Examining the nutritional quality of Canadian packaged foods and beverages with and without nutrition claims. *Nutrients*. 10:832. 10.3390/nu10070832 29954102 PMC6073495

[B20] Garretson J. A. Burton S. (2000). Effects of nutrition facts panel values, nutrition claims, and health claims on consumer attitudes, perceptions of disease-related risks, and trust. *J. Public Policy Mark*. 19 213–227. 10.1509/jppm.19.2.213.17133 11670861

[B21] Gounaris S. P. Tzempelikos N. A. Chatzipanagiotou K. (2007). The relationships of customer-perceived value, satisfaction, loyalty and behavioral intentions. *J. Relatsh. Mark.* 6 63–87. 10.1300/J366v06n01_05

[B22] Graessley S. Horak J. Kovacova M. Valaskova K. Poliak M. (2019). Consumer attitudes and behaviors in the technology-driven sharing economy: motivations for participating in collaborative consumption. *J. Self-Gov. Manag. Econ.* 7 25–30. 10.22381/JSME7120194

[B23] Graham D. J. Roberto C. A. (2016). Evaluating the impact of U.S. food and drug administration–proposed nutrition facts label changes on young adults’ visual attention and purchase intentions. *Health Educ. Behav.* 43 389–398. 10.1177/1090198116651082 27230269

[B24] Hollowell J. C. Rowland Z. Kliestik T. Kliestikova J. Dengov V. V. (2019). Customer loyalty in the sharing economy platforms: how digital personal reputation and feedback systems facilitate interaction and trust between strangers. *J. Self-Gov. Manag. Econ.* 7 13–18. 10.22381/jsme7120192

[B25] Kaiser H. F. (1974). An index of factorial simplicity. *Psychometrika* 39 31–36. 10.1007/BF02291575

[B26] Katona G. (1967). What is consumer psychology? *Am. Psychol*. 22 219–226. 10.1037/h0037767 6044876

[B27] Kim H. John D. R. (2008). Consumer response to brand extensions: construal level as a moderator of the importance of perceived fit. *J. Consum.Psychol.* 18 116–126. 10.1016/j.jcps.2008.01.006

[B28] Kline R. B. (2005). *Principles and Practice of Structural Equation Modeling*, 2nd Edn. New York, NY: Guilford Press.

[B29] Klopčič M. Slokan P. Erjavec K. (2019). Consumer preference for nutrition and health claims: a multi-methodological approach. *Food Qual. Prefer.* 82:103863. 10.1016/j.foodqual.2019.103863

[B30] Kotler P. Armstrong G. (2011). *Principle of Marketing*, 14th Edn. Hoboken, NJ: Prentice Hall.

[B31] Lei P. Wu Q. (2007). Introduction to structural equation modeling: issues and practical considerations. *Educ. Meas. Issues Pract.* 26 33–43. 10.1111/j.1745-3992.2007.00099.x

[B32] Loureiro M. L. Mccluskey J. J. (2000). Assessing consumer response to protected geographical identification labeling. *Agribusiness* 16 309–320. 10.1002/1520-6297(200022)16:3<309::AID-AGR4<3.0.CO;2-G

[B33] Mathios A. D. (1998). The importance of nutrition labeling and health claim regulation on product choice: an analysis of the cooking oils market. *Agric. Res. Econ. Rev*. 27 159–168. 10.1017/S1068280500006481

[B34] Meilhan D. (2019). Customer value co-creation behavior in the online platform economy. *J. Self-Gov. Manag. Econ.* 7 19–24. 10.22381/JSME7120193

[B35] Ministry of Health of the PRC (2011). *General Rules for Nutrition Labeling of Pre-Packaged Food*. GB28050-2011. Beijing: Ministry of Health of the PRC.

[B36] Miricǎ C.-O. (2019). The behavioral economics of decision making: explaining consumer choice in terms of neural events. *Econ. Manag. Financ. Mark.* 14 16–20. 10.22381/EMFM14120192

[B37] Neale M. C. Hunter M. D. Pritikin J. N. Zahery M. Brick T. R. Kirkpatrick R. M. (2016). OpenMx20: extended structural equation and statistical modeling. *Psychometrika* 81 535–549. 25622929 10.1007/s11336-014-9435-8 PMC4516707

[B38] Nunnally J. C. (1978). *Psychometric Theory.* New York, NY: Mcgraw-Hill.

[B39] O’Neil P. M. Jarrell M. P. (1992). Psychological aspects of obesity and very-low-calorie diets. *Am. J. Clin. Nutr.* 56 185S–189S. 10.1111/j.1747-0080.2009.01380.x 1615881

[B40] Oostenbach L. H. Slits E. Robinson E. Sacks G. (2019). Systematic review of the impact of nutrition claims related to fat, sugar and energy content on food choices and energy intake. *BMC Public Health* 19:1296. 10.1186/s12889-019-7622-3 31615458 PMC6794740

[B41] Pocol C. B. Marinescu V. Dabija D.-C. Amuza A. (2021). Clustering generation Z university students based on daily fruit and vegetable consumption: empirical research in an emerging market. *Br. Food J.* 4 47–53. 10.1108/BFJ-10-2020-0900

[B42] Popescu G. H. Ciurlǎu F. C. (2019). Making decisions in collaborative consumption: digital trust and reputation systems in the sharing economy. *J. Self-Gov. Manag. Econ.* 7 7–12. 10.22381/JSME7120191

[B43] Priester J. R. (2010). The use of structural equation models in consumer psychology: a methodological dialogue on its contributions, cautions, and concerns. *J. Consum. Psychol.* 20 205–207. 10.1016/j.jcps.2010.03.005

[B44] Reibstein D. J. Youngblood S. A. Fromkin H. L. (1975). Number of choices and perceived decision freedom as a determinant of satisfaction and consumer behavior. *J. Appl. Psychol.* 60 434–437. 10.1037/h0076906

[B45] Song J. Huang J. Chen Y. Zhu Y. Li H. Wen Y. (2015). The understanding, attitude and use of nutrition label among consumers (China). *Nutr. Hosp*. 31 2703–2710. 10.3305/nh.2015.31.6.8791 26040385

[B46] Story M. French S. (2004). Food advertising and marketing directed at children and adolescents in the US. *Int. J. Behav. Nutr. Phys. Act*. 1:3. 10.1186/1479-5868-1-3 15171786 PMC416565

[B47] Stranieri S. Baldi L. Banterle A. (2010). Do nutrition claims matter to consumers? An empirical analysis considering European requirements. *J. Agric. Econ.* 61 15–33. 10.1111/j.1477-9552.2009.00223.x

[B48] Thompson B. (2004). *Exploratory and Confirmatory Factor Analysis: Understanding Concepts and Applications.* Washington, DC: American Psychological Association.

[B49] Verbeke W. Scholderer J. Lähteenmäki L. (2009). Consumer appeal of nutrition and health claims in three existing product concepts. *Appetite* 52 684–692. 10.1016/j.appet.2009.03.007 19501767

[B50] Wang S. Chen Y. Liu M. Hong Z. Sun D. Du Y. (2011). The changes of nutrition labeling of packaged food in Hangzhou in China during 2008~2010. *PLoS One* 6:e28443. 10.1371/journal.pone.0028443 22194836 PMC3237440

[B51] Wezemael L. V. Caputo V. Nayga R. M. Chryssochoidis G. Verbeke W. (2014). European consumer preferences for beef with nutrition and health claims: a multi-country investigation using discrete choice experiments. *Food Policy* 44 167–176. 10.1016/j.foodpol.2013.11.006

[B52] Wing R. R. Marcus M. D. Blair E. H. Burton L. R. (1991). Psychological responses of obese type II diabetic subjects to very-low-calorie diet. *Diabetes Care* 14 596–599. 10.2337/diacare.14.7.596 1914801

[B53] Zhang X. Dagevos H. He Y. Lans I. V. D. Zhai F. (2008). Consumption and corpulence in China: a consumer segmentation study based on the food perspective. *Food Policy* 33 37–47.

