# Supplementary material for: Effects of Low-Calorie Nutrition Claim on Consumption of Packaged Food in China: An Application of the Model of Consumer Behavior
Source: Front Psychol. 2022 Jan 28;12:799802. doi: 10.3389/fpsyg.2021.799802 (PMC8833154; doi:10.3389/fpsyg.2021.799802)
Supplement: Supplementary file 1 [file Data_Sheet_1.doc]

**Supplementary materials :** The questionnaire

**Section 1 Survey questions about marketing stimuli**

Q1-1 I have ever seen food with low calorie nutrition claim on sales.

A.strongly disagree B. disagree C. neither agree nor disagree D.agree E.strongly agree

Q1-2 I know that the price of food with low calorie nutrition claim is affordable.

A.strongly disagree B. disagree C. neither agree nor disagree D.agree E.strongly agree

Q1-3 I have ever seen food with low calorie nutrition claim sold in many places.

A.strongly disagree B. disagree C. neither agree nor disagree D.agree E.strongly agree

Q1-4 I have ever seen the food with low calorie nutrition claim on promotion.

A. strongly disagree B. disagree C. neither agree nor disagree D.agree E.strongly agree

**Section 2 Survey questions about consumer psychology**

Q2-1 I would follow my friends and relatives’ example if they all read low calorie nutrition claim when shopping.

A.strongly disagree B. disagree C. neither agree nor disagree D.agree E.strongly agree

Q2-2 I would read low calorie nutrition claim even if none of my friends and relatives did it when shopping.

A.strongly disagree B. disagree C. neither agree nor disagree D.agree E.strongly agree

Q2-3 I would buy the food with low calorie nutrition claim which is beyond my factual income.

A.strongly disagree B. disagree C. neither agree nor disagree D.agree E.strongly agree

Q2-4 I would pay attention to the actual benefits of food with low calorie nutrition claim.

A.strongly disagree B. disagree C. neither agree nor disagree D.agree E.strongly agree

**Section 3 Survey questions about consumer decision making**

Q3-1 I believe low calorie nutrition claim help make healthy food choice.

A.strongly disagree B. disagree C. neither agree nor disagree D.agree E.strongly agree

Q3-2I believe low calorie nutrition claim help understand nutritional properties of food.

A.strongly disagree B. disagree C. neither agree nor disagree D.agree E.strongly agree

Q3-3 I have ever read low calorie nutrition claim when shopping.

A.strongly disagree B. disagree C. neither agree nor disagree D.agree E.strongly agree

Q3-4I have bought foods with low calorie nutrition claim when shopping.

A.strongly disagree B. disagree C. neither agree nor disagree D.agree E.strongly agree

**Section 4 Survey questions about consumer responses**

Q4-1I have ever made choices among different kinds of foods through low calorie nutrition claim.

A.strongly disagree B. disagree C. neither agree nor disagree D.agree E.strongly agree

Q4-2I have ever made choices among different brands of similar foods through low calorie nutrition claim.

A.strongly disagree B. disagree C. neither agree nor disagree D.agree E.strongly agree

Q4-3 I have ever seized the moment to buy foods with low calorie nutrition claim.

A.strongly disagree B. disagree C. neither agree nor disagree D.agree E.strongly agree

Q4-4I have ever made choices among different amounts of food through low calorie nutrition claim.

A.strongly disagree B. disagree C. neither agree nor disagree D.agree E.strongly agree

**Section 5 Demographic characteristics**

Q5-1 Your gender : A. Male B. Female

Q5-2 Your age: A. under 18 years old B. 18-44 years old C.45-59 years old D. 60 years old and above

Q5-3 Your education level: A. Primary school and below B. Junior high school C. High school

D. Junior college or undergraduate E. Postgraduate or above

Q5-4 Your individual annual income on average (Yuan):

A.<10,000 B.10,000-50,000 C.50,001-100,000 D.100,001-150,000 E.150,001-200,000

F.>200,001
